# 

**Published:** 1890-07

**Authors:** 


### Invitation to the Tenth International Medical Congress.

In accordance with the decision of the Ninth Congress at Washington, the Tenth International Medical Congress will be held at Berlin from the 4th to the 9th of August, 1890.

By the delegates of the German Medical Faculties and the chief medical societies of the German Empire the undersigned have been appointed members of the General Committee of Organization. A Special Committee of Organization has also been appointed for each of the different sections to arrange the scientific problems to be discussed at the meetings of the respective sections. An International Medical and Scientific Exhibition will also be held by the congress.

We have the honor to inform you of the above decisions, and at the same time cordially to invite your attendance at the congress. We should esteem it a favor if you would kindly extend this invitation to your friends in medical circles as opportunity may offer.

We beg to accompany our invitation by a copy of the statutes and programme, as also by a list of the intended sections and their special committees of organization. Dr. Rudolf Virchow, President; Dr. Von Bergmann, Dr. Leyden, Dr. Waldeyer, Vice-Presidents; Dr, Lassar, Secretary General.

All communications must be directed to the General Secretary, Berlin NW., Karlstrasse 19.

SECTION XIV.—DENTAL AND ORAL SECTION.

There will be five general subjects for discussion as follows:

1. Bromide of Ethyl Narcosis.
2. Pyorrhœa Alveolaris.
3. Micro-Organisms in Caries.
4. Crown and Bridge Work.
5. Irregularities of the Teeth.

Of these one has been assigned to Germany, one to England, one to France, and two to America.

Dr. W. C. Barrett, of Buffalo, N. Y., has been designated by the General Committee to present the subject of Crown and Bridge Work and Dr. E. S. Talbot, that of Irregularities of the Teeth. These five subjects with the clinics and demonstrations, by men selected by the General Committee, will form the official programme of the section.

The other papers read will be selected from the voluntary contributions and will be as many as time and the General Committee will permit.

The American officers of the section consists of four honorary presidents as follows: W. C. Barrett, M.D., D.D.S., Buffalo, N. Y.; J. Taft, M.D., D.D.S., Cincinnati, Ohio; E. S. Talbott, M.D., D.D.S., Chicago, Ill.; H. J. McKellops, D.D.S., St. Louis, Mo.

R. R. Andrews, D.D.S., of Cambridge, Mass., has been selected by the board as Honorary Secretary.

A large number of American dentists have already signified their intention to be present at the congress.

---

A DREADFUL TOOTHACHE.—Men will do almost any thing when suffering from toothache. M. X. was one day attacked on a railroad train and suffered the wildest tortures. "Ah!" cried he to one of his friends who was traveling with him, "if I only had a piece of cotton for my tooth; but unfortunately"—here he uttered a cry of joy. "Saved! saved!" and pulled some cotton from his neighbor's ear.

### Notices.

MEETING OF THE AMERICAN DENTAL ASSOCIATION.—The railroad arrangements are not all completed, but enough is known to assure at least the usual reduction of one and a third fare on certificate plan, by all the different passenger associations.

Arrangements are being made for a special train from Chicago which will leave here Sunday afternoon and reach Excelsior Springs Monday morning. This will give the entire day, Monday, for the different sections to complete their reports. All parties wishing to go on this special train will confer a favor by letting us know at once so that we may know how many to arrange for. Just what rate will be secured for round trip is not definitely settled, but we expect a low one. A notice will be issued later giving exact time of starting and route selected.

Application has been made for reduced rates for the four associations: The American Dental Association, College Faculty Association, National Board of Dental Examiners and the Dental Protective Association. We are trying to get this rate good for ten days so that we need not adjourn until everything is finished.

Parties purchasing tickets should be sure to get receipt showing that they have paid full fare going which will enable them to get return ticket for one third regular fare. J. N. CROUSE,

Chr. Ex. Com., 2231 Prairie Ave., Chicago.

### The Dental Register,

A Monthly Magazine Devoted to the Interests of the

**DENTAL PROFESSION.**

*Terms Reduced to $2.00 Per Year. Single Copies, 25 Cents.*

**EDITED BY PROF. J. TAFT, D.D.S.**

—AND—

**Published by SAM'L A. CROCKER & CO., Ohio Dental and Surgical Depot.**

The volume commences in January and closes in December.

The Register being issued monthly is always fresh, therefore Indispensable to the practitioner who desires to keep posted up with the new inventions and improvements which are daily developed. Neither expense nor effort will be spared to give value and variety to its contents. Articles will be illustrated with well executed engravings whenever they will add to the interest or assist to explanation.

Its extensive circulation and frequency of issue make it one of the BEST DENTAL ADVERTISING MEDIUMS in the United States.

All subscriptions must begin with January or July.

Local or special notices, five lines or less, will be inserted for $3 each insertion; over five lines, 50 cts. per line.

All advertising bills payable in advance, or at furthest, after the issue of the first number containing the advertisement. All transient advertisements to be paid for in advance.

☞CONTRIBUTIONS SOLICITED.

Exclusively Editorial Communications should be addressed to the Editor.

The latest number of the Register may be ordered with or without other goods at any time, and all letters should be addressed to

**SAM'L A. CROCKER & CO., Ohio Dental and Surgical Depot, Cincinnati, O.**

